# Probing the Cardiovascular Toxic Effects of Long-Term Exposure to Dibutyl Phthalate in Sprague-Dawley Rats Based on Oxidative Inflammation and Metabolic Pathways: Implications for the Heart and Blood Vessel

**DOI:** 10.3390/toxics13100815

**Published:** 2025-09-25

**Authors:** Xiao Liang, Qi Huang, Yang Wu, Deyu Zhu, Zhuangzhuang Wei, Qing Feng, Ping Ma, Xu Yang, Cuiyu Bao, Xinyu Bao

**Affiliations:** 1 Key Laboratory of Environmental Related Diseases and One Health, Xianning Medical College, Hubei University of Science and Technology, Xianning 437100, Chinayangxu@mail.ccnu.edu.cn (X.Y.);; 2 School of Public Health and Nursing, Hubei University of Science and Technology, Xianning 437100, China; 3 Centre for Biological Science and Technology, Key Laboratory of Cell Proliferation and Regulation Biology of Ministry of Education, Faculty of Arts and Sciences, Beijing Normal University, Zhuhai 519000, China; 4 Institute of Natural Antioxidants and Antioxidant Inflammation, Dali University, Dali 671003, China

**Keywords:** dibutyl phthalate, cardiotoxicity, vascular toxicity, oxidative stress, pyroptosis, metabonomics

## Abstract

Background: Dibutyl phthalate (DBP) is a prevalent environmental pollutant that can accumulate in organisms, becoming amplified after the food cycle and ultimately affecting human health. Recent studies have provided evidence suggesting a potential association between exposure to DBP and cardiovascular diseases (CVDs). Objectives: This study’s objective is to investigate the toxic cardiovascular effects of long-term exposure to DBP, particularly its impact on the heart and blood vessels. To be specific, we hypothesized and verified the potential mechanisms underlying DBP-induced cardiac and vascular injuries, focusing on oxidative stress, pyroptosis, inflammatory responses, and metabolic pathways. Methods: The rats were divided into 5 groups: Control group, DBP-Low group, DBP-Medium group, DBP-High group, and DBP-High + Vitamin E group. The entire experimental period lasted 12 weeks. We conducted examinations on echocardiography, histopathology, oxidative stress biomarkers, pyroptosis-related biomarkers, and inflammatory cytokine biomarkers. Additionally, we carried out serum metabolomics analysis. Result: Our research findings indicate that long-term exposure to DBP can cause significant toxic effects on the cardiovascular system. Specifically, DBP leads to changes in oxidative stress indicators (ROS and an increase in MDA levels, alongside a decrease in GSH levels) and protein levels related to pyroptosis (NLRP3, Caspase-1 and GSDMD levels increase) in cardiac and vascular tissues, triggering oxidative inflammatory responses (IL-1β and IL-18 levels increase), damaging the heart and blood vessels (organizational structure deformation and collagen fiber infiltration) and ultimately affecting their functions (abnormalities in cardiac function and hemodynamics). Additionally, the results of metabolomics studies suggest that metabolic pathways (Biotin metabolism, TCA cycle, Vitamin B6 metabolism, Pantothenate and CoA biosynthesis, and Riboflavin metabolism) and metabolites may also be of great significance. Conclusion: Long-term exposure to DBP can induce cardiovascular toxicity in rats, manifesting as cardiac and vascular damage, as well as alterations in organ function. This process is characterized by oxidative stress, activation of the pyroptosis pathway, inflammatory responses, and modifications to metabolic pathways.

## 1. Introduction

Phthalates (PAEs), recognized as the most widely produced and consumed plasticizers globally, are extensively utilized in various fields, including building decoration, food packaging, medical devices, and personal care products. These materials are attached to plastic surfaces via non-covalent connections, facilitating their straightforward release into various environmental mediums, such as air, water, and soil, which contributes to their categorization as widespread environmental contaminants [1]. Among the different PAEs, di-n-butyl phthalate (DBP) is especially common. As a low-molecular-weight phthalate, DBP can typically be detected in both indoor and outdoor settings and may enter the human body through inhalation, ingestion, or dermal exposure [2]. Research indicates that DBP exposure is linked to endocrine disruption, immune modulation, and oxidative inflammation; however, its toxic effects on the cardiovascular system remain not fully understood [3,4,5].

Cardiovascular diseases (CVD) are one of the leading causes of death worldwide. The World Health Organization (WHO) reports that approximately 18 million people die from these diseases each year, accounting for over 30% of global deaths. In 2019, about 17.9 million people worldwide died from cardiovascular diseases, representing 32% of the total global deaths. Predictions indicate that by 2050, the number of deaths caused by cardiovascular diseases is expected to reach 35.6 million, with a mortality rate increase of 73.4% [6]. Among them, cardiovascular diseases account for 40% of the total deaths in the Chinese population [7]. Epidemiological studies have established a correlation between phthalate exposure and the incidence of cardiovascular diseases [8]. Additionally, experimental research has demonstrated that exposure to DBP can lead to damage to cardiac tissues and blood vessels [9,10].

Pyroptosis is an inflammatory form of cell death that is closely associated with oxidative stress. ROS generated by oxidative stress can stimulate the activation of NLRP3, which mediates the occurrence of pyroptosis, ultimately leading to the release of inflammatory factors, such as IL-1β and IL-18, and resulting in oxidative inflammation [11]. Notably, studies have demonstrated that ROS-mediated pyroptosis-induced oxidative inflammation plays a crucial role in cardiovascular diseases [12]. Additionally, our preliminary studies indicate that DBP exposure is significantly linked to oxidative stress [13,14,15]. Therefore, DBP may contribute to the development of cardiovascular diseases, including damage to the heart and blood vessels, through the mechanism of oxidative inflammation.

Metabolomics is an innovative omics technology that encompasses the qualitative and quantitative analysis of small-molecule metabolites within biological organisms or cells. Its primary objective is to elucidate the relationships between metabolites and physiological or pathological changes [16]. Numerous studies have demonstrated a strong correlation between metabolic pathways and the onset of cardiovascular diseases [17]. Furthermore, our preliminary experimental research indicates that exposure to DBP can significantly alter metabolic pathways [14,15]. Consequently, DBP may contribute to heart and vascular damage, thereby increasing the risk of cardiovascular diseases through its influence on relevant metabolic pathways.

**Figure 1:**
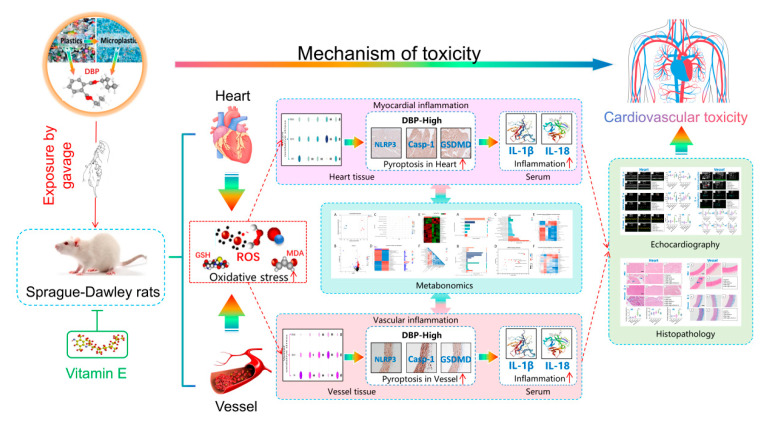
Mechanisms of DBP-induced cardiac and vascular injury and the protective role of vitamin E.

Based on our previous research and the potential etiological factors discussed in the aforementioned experiments, we propose the following scientific hypotheses (Figure 1): Long-term exposure to DBP may lead to damage in the heart and blood vessels of SD rats, potentially involving mechanisms such as oxidative stress, pyroptosis, inflammatory responses, and alterations in metabolic pathways. Furthermore, vitamin E, recognized as a prominent antioxidant, has the potential to mitigate heart and vascular damage induced by oxidative stress.

## 2. Materials and Methods

### 2.1. Experimental Animals

SPF-grade SD male rats, weighing approximately 180 to 200 g, were acquired from the Hubei Provincial Center for Experimental Animals in Wuhan, China. After purchase, these rats were given a 1-week period of acclimatization feeding and were provided with feed and water, which they could access freely. Throughout the entire experimental period, the rats were continuously kept in appropriate room temperature, humidity, and lighting conditions. All procedures received approval from the Research Management Office of Hubei University of Science and Technology, and the approval certificate can be provided upon request (ID: HBUST-IACUC-2023-026).

### 2.2. Reagents and Kits

DBP (CAS: 84-74-2) and Vitamin E (CAS: 59-02-9) were sourced from Sigma-Aldrich (St. Louis, MO, USA). The kit for rat GSH (glutathione) was provided by Nanjing Biotech Co., Ltd. located in Nanjing, China. Moreover, rat ELISA kits for measuring IL-1β (Interleukin-1β) and IL-18 (Interleukin-18) were obtained from Shanghai Enzyme-Linked Biotech Co., Ltd. based in Shanghai, China. All other reagents used throughout this research met the highest commercial quality or adhered to defined standards.

### 2.3. Experimental Protocol

**Figure 2:**
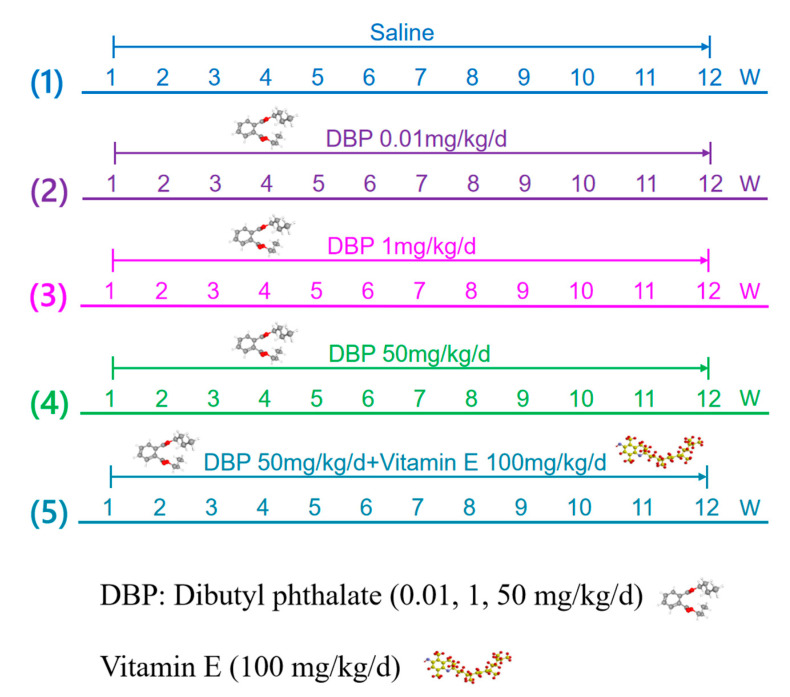
Experimental protocol.

We divided 40 rats into 5 groups, with 8 rats in each group. and provided with a commercial diet and filtered water. We randomly divided the SD rats into 5 groups, as shown in Table 1 and Figure 2. The specific method of grouping is as follows: (1) Control: Replace DBP exposure with normal saline; (2) DBP-Low: 0.01 mg/kg/day DBP exposure; (3) DBP-Medium: 1.0 mg/kg/day DBP exposure; (4) DBP-High: 50 mg/kg/day DBP exposure; (5) DBP-High + VitE: 50 mg/kg/day DBP exposure + 100 mg/kg/day vitamin E treatment; Group (1) served as the blank control group. Groups (2), (3), and (4) comprised the low-, medium-, and high-dose DBP exposure groups, respectively, aimed at investigating the cardiovascular toxicity induced by varying concentrations of DBP. Groups (5) represented the antagonist groups, which were designed to explore the protective effects of vitamin E against the cardiovascular toxicity induced by DBP exposure.

We employed the most common exposure method by orally administering DBP. Based on the tolerable daily intake of DBP established by the European Food Safety Authority at 0.01 mg/kg/d [18], we selected low, medium, and high exposure concentrations of 0.01, 1, and 50 mg/kg/d, respectively. The selection of the high-dose (50 mg/kg/day) for DBP was based on our previous experimental article [2,13,19,20]. In order to counteract the toxic effects of DBP, we utilized the well-known antioxidant vitamin E as an antagonist at a concentration of 100 mg/kg/d. The concentration of vitamin E was determined based on findings from prior studies [21,22]. The entire experimental period lasted for 12 weeks, aiming to investigate the long-term effects of DBP exposure.

### 2.4. Echocardiography Evaluation

After the last administration of the drug, rats were anesthetized using inhalation methods (isoflurane, 4% induction, 2% maintenance). A high-frequency ultrasound imaging system (Vevo 2100, VisualSonics Company, Toronto, ON, Canada) with an MS250 (20 MHz, VisualSonics Company) transducer probe was utilized to conduct the echocardiography. Parameters such as aortic acceleration time (AAT), aortic ejection time (AET), AAT/AET ratio, peak velocity (Peak Vel), peak gradient (Peak Grad), and velocity time integral (VTI) were determined through blood flow spectrum analysis. Moreover, the assessment of LVEF and LVFS were evaluated by analyzing M-mode ultrasound images. In addition, the ultrasound images of the PWD-mode and the TD-mode were evaluated to obtain the E/A ratio, the E/E’ ratio, DT, and MPI. At the end of the experiments, rats were euthanized by cervical dislocation under inhalation of 5% isoflurane deep anaesthesia.

### 2.5. Histopathological and Immunohistochemical Analysis

We randomly selected 6 tissue samples (Three hearts and three blood vessels) from each group of rats, and each tissue sample was subjected to both histopathological examination and immunohistochemical analysis. We collected the heart and vascular tissues, and then fixed them using a 4% polyformaldehyde solution. To prepare the tissue samples for analysis, we followed a step of sequentially immersing them in different concentrations of alcohol and xylene. Finally, the tissues were embedded in paraffin according to the traditional method. After 24 h, 4-micron thick sections were cut, dewaxed, and fixed on glass slides. Following that, H&E staining, Masson staining, and immunohistochemical tests were carried out. The immunohistochemical evaluation was performed subsequent to the extraction of antigens and the inactivation of internal enzymes, in accordance with the techniques employed in our earlier studies [2]. The analysis of the aortic tissue sections revealed the intima-media thickness located beneath the intima [23].

### 2.6. ROS, MDA and GSH Assay

Heart and vascular tissues were processed through homogenization with a glass homogenizer in ice-cold phosphate-buffered saline (PBS, pH 7.5) at a volume ratio of 10 mL for each gram of tissue. The mixture was subsequently centrifuged at 10,000 RPM for 10 min, and the supernatant obtained was collected. The concentrations of ROS in the supernatants from the heart and vascular tissues were assessed using the universal ROS indicator (DCFH-DA) in accordance with a previously established protocol [24]. MDA is measured using TBA method [25]. GSH is measured using the accompanying kit and in accordance with the manufacturer’s instructions.

### 2.7. IL-1β and IL-18 ELISA Assay

The serum levels of IL-1β and IL-18 were assessed using ELISA kits sourced from Shanghai Enzyme-linked Biotechnology Co., Ltd. based in Shanghai, China. The measurements were performed following the guidelines provided by the manufacturer, with the sensitivities of the IL-1β and IL-18 kits being 1.0 pg/mL.

### 2.8. Untargeted Metabolomics Analysis of Rat Serum

Regarding the specific procedures of metabolomics, we primarily adhere to our previously established experimental methods [14,15]. Serum samples are prepared in accordance with standard protocols, and detection is conducted using LC-MS. For metabolomics, due to limitations in resources and other factors, a sample size of *n* = 4 was ultimately adopted. The determination of this sample size was based on the generally accepted standards in this field for similar studies [26,27,28]. The specific sample preparation, instruments and data analysis methods are included in the Supplementary Materials. We have provided full identification and quantification details as Supplementary Material.

### 2.9. Statistical Analysis

The data’s statistical analysis and graph creation were performed using GraphPad Prism 10 software, with results expressed as mean ± standard deviation (SD). To assess the statistical differences between the groups, a one-way analysis of variance (ANOVA) was utilized. For comparing the differences between each dose group and the control group, the least significant difference (LSD) method was applied. *p* < 0.05 was deemed to be statistically significant. In the statistical analysis of metabolomics, metabolites exhibiting VIP > 1, as determined by the OPLS-DA model, along with *p* < 0.05 from the *t*-test, were recognized as significantly different metabolites.

## 3. Results

### 3.1. Long-Term Exposure to DBP Can Cause Heart and Vascular Dysfunction in SD Rats

**Figure 3:**
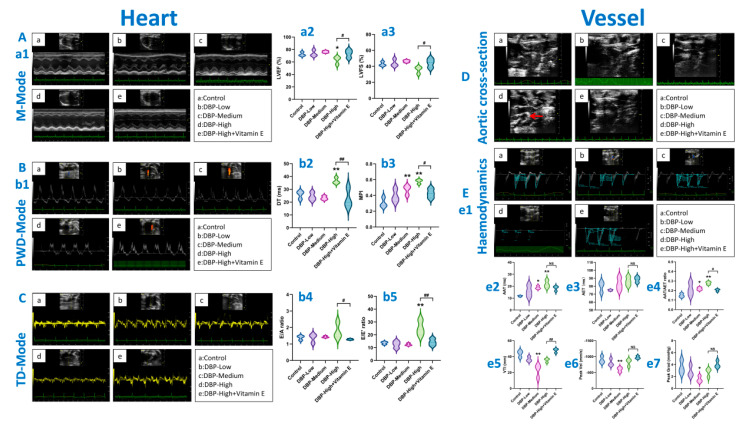
Echocardiography evaluation. (**A**) (**a1**): M-Mode, (**a2**): LVEF, (**a3**): LVFS. (**B**) (**b1**): Pulsed-Wave Doppler Mode, (**b2**): DT, (**b3**): MPI, (**b4**): E/A, (**b5**): E/E’. (**C**) Tissue Doppler Mode. (**D**) Aortic arch cross-section, lesion region (red arrows). (**E**) (**e1**): Haemodynamics of the aortic arch, (**e2**): AAT, (**e3**): AET, (**e4**): AAT/AET, (**e5**): VTI, (**e6**): Peak Vel, (**e7**): Peak Grad. *: *p* < 0.05, **: *p* < 0.01, compared with the Control group; #: *p* < 0.05, ##: *p* < 0.01, compared with the DBP-High group; NS: *p* > 0.05. *n* = 3.

For echocardiography, due to limitations such as sample availability and resources, a sample size of *n* = 3 was ultimately adopted. The determination of this sample size was based on the generally accepted standards in this field for similar studies [29,30,31]. The echocardiographic assessment (Figure 3) revealed the following findings: M-Mode (Figure 3A) demonstrated that the LVEF level (Figure 3(a2)) in the DBP-High group was significantly lower than that in the Control group. PWD-Mode (Figure 3B) and TD-Mode (Figure 3C) indicated that the MPI levels (Figure 3(b3)) in both the DBP-Medium and DBP-High groups were significantly higher than those in the Control group. Additionally, the DT level (Figure 3(b2)) and E/E’ level (Figure 3(b5)) in the DBP-High group were also significantly higher than those in the Control group. These results suggest that long-term exposure to high-doses of DBP may lead to heart dysfunction. However, after treatment with vitamin E, the cardiac dysfunction in the DBP-High group was significantly improved (Figure 3(a2,a3,b2–b5)).

Additionally, the aortic cross-section (Figure 3D) revealed a significant enhancement in the echo of the aortic arch wall in the DBP-High group compared to the Control group, indicating that long-term high-dose DBP exposure may lead to damage in the aortic wall. Haemodynamics (Figure 3E) showed that both the DBP-Medium and DBP-High groups exhibited significantly elevated levels of AAT (Figure 3(e2)) and AAT/AET (Figure 3(e4)) compared to the Control group. Meanwhile, the levels of VTI, Peak Vel, and Peak Grads in the DBP-Medium group were also significantly lower than those in the Control group (Figure 3(e5–e7)). These results indicate that both medium-dose and high-dose DBP exposure over the long term may induce vascular dysfunction, thereby leading to hemodynamic abnormalities. However, upon treatment with vitamin E, the hemodynamic conditions in the DBP-High group significantly improved (Figure 3(e4,e5)).

### 3.2. Long-Term Exposure to DBP Can Cause Histopathological Damage to the Heart and Blood Vessels in SD Rats

**Figure 4:**
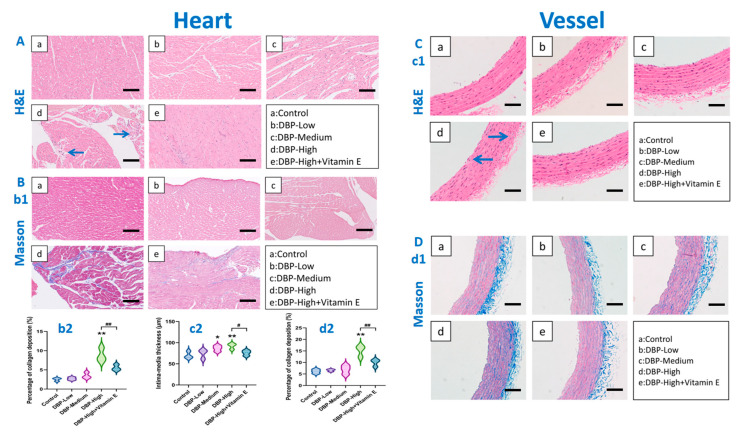
Histopathology analysis. The following images are representative images. (**A**) H&E staining of cardiac: Histologic section (20 X, scale bar 100 µm), area of lesion (marked by blue arrow). (**B**) Masson staining of cardiac, (**b1**): Histologic section (20 X, scale bar 100 µm); (**b2**): Deposition percentage of collagen. (**C**) H&E staining of blood vessel, (**c1**): Histologic section (20 X, scale bar 100 µm), region of lesion (marked with blue arrow); (**c2**): Thickness of intima-media. (**D**) Masson staining of blood vessel, (**d1**): Histologic section (20 X, scale bar 100 µm); (**d2**): Deposition percentage of collagen. *: *p* < 0.05, **: *p* < 0.01, compared with the Control group; #: *p* < 0.05, ##: *p* < 0.01, compared with the DBP-High group; NS: *p* > 0.05. *n* = 3.

The histopathological evaluation results (Figure 4) indicate that H&E staining of the heart (Figure 4A) revealed myocardial interstitial inflammatory infiltration in the DBP-High group compared to the Control group. This finding suggests that long-term high-dose exposure to DBP leads to myocardial injury. Additionally, Masson staining results (Figure 4B) corroborate these findings, showing that the area of collagen deposition in the DBP-High were significantly greater than that in Control (Figure 4(b2)). This indicates that prolonged high-dose DBP exposure may result in both myocardial tissue damage and subsequent repair processes. However, following treatment with vitamin E, significant improvement in cardiac tissue lesions was observed in the DBP-High group (Figure 4(c2,d2)).

Furthermore, the results of vascular H&E staining (Figure 4C) indicated that, compared to the Control group, the intima-media thickness was significantly increased in the DBP-Medium and DBP-High groups (Figure 4(c2)). Additionally, the vascular endothelial cells in the DBP-High group exhibited disorganized arrangement and inflammatory infiltration, suggesting that long-term high-dose DBP exposure leads to vascular damage. Meanwhile, the results of Masson staining (Figure 4D) were consistent with these findings, showing that the collagen deposition area in the DBP-High group was significantly greater than that in the Control group (Figure 4(b2)), indicating that long-term high-dose DBP exposure can cause both damage and repair in vascular tissues. However, following vitamin E treatment, the vascular tissue lesions in the DBP-High group showed significant improvement (Figure 4(b2)).

### 3.3. Long-Term Exposure to DBP Leads to Oxidative Stress in the Cardiac and Blood Vessels of SD Rats

**Figure 5:**
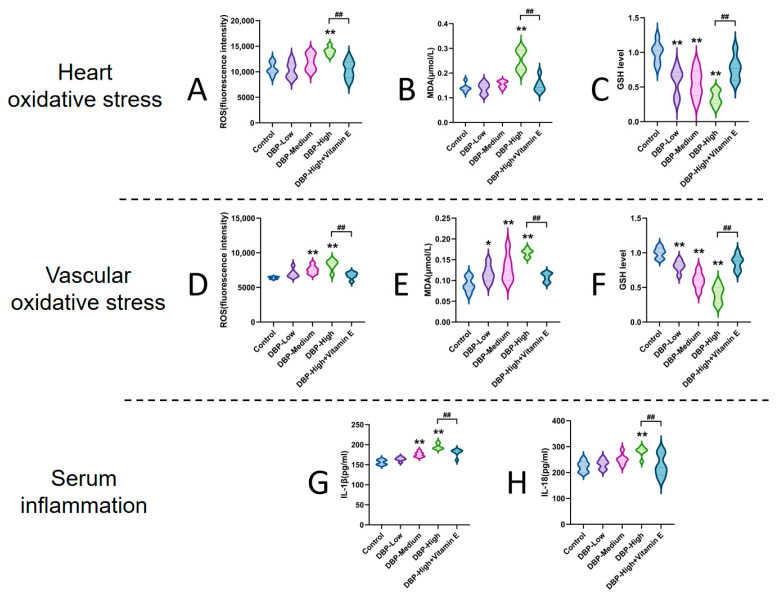
Oxidative stress and inflammation: (**A**) ROS levels in heart; (**B**) MDA levels in heart; (**C**) GSH levels in heart. (**D**) ROS levels in blood vessel; (**E**) MDA levels in blood vessel; (**F**) GSH levels in blood vessel. (**G**) IL-1β levels in serum; (**H**) IL-18 levels in serum. *: *p* < 0.05, **: *p* < 0.01, compared with the Control group; ##: *p* < 0.01, compared with the DBP-High group; NS: *p* > 0.05. *n* = 6.

The indicators of oxidative stress and inflammatory factors (Figure 5) demonstrated that, compared to the Control group, the levels of ROS and MDA in the heart were significantly elevated (132.5% and 178.6%) in the DBP-High group, while the levels of GSH were markedly reduced (57.9%, 55.2% and 34%) in the DBP-Low, DBP-Medium, and DBP-High groups. Additionally, in the blood vessels, the levels of ROS and MDA were significantly increased (118.3% and 144.4%) in the DBP-Medium groups when compared to the Control group, alongside a significant decrease (59.8%) in GSH levels. Meanwhile, the levels of ROS and MDA were significantly increased (129.3% and 185.6%) in the DBP-High groups when compared to the Control group, alongside a significant decrease (40.5%) in GSH levels. Furthermore, the levels of IL-1β were significantly elevated (112.8% and 124.9%) in the DBP-Medium and DBP-High groups compared to the Control group, with IL-18 levels also significantly increased (125.2%) in the DBP-High group. However, treatment with vitamin E led to significant improvements in oxidative stress and inflammation in the DBP-High group. These results suggest that long-term exposure to DBP can induce oxidative inflammation in the heart and blood vessels, with effects becoming more pronounced at higher doses of DBP. Vitamin E can mitigate these conditions through its antioxidant properties.

### 3.4. Long-Term Exposure to DBP Can Induce Oxidative Inflammation in the Heart and Blood Vessels of SD Rats Through ROS-Mediated Pyroptosis

**Figure 6:**
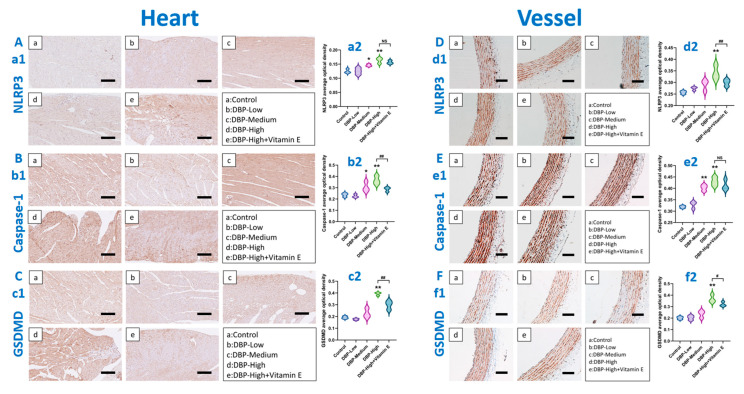
Immunohistochemistry of pyroptosis. The following images are representative images. (**A**) (**a1**): NLRP3 immunohistochemical staining in cardiac tissue (20 X, scale bar 100 µm), (**a2**): Mean optical density measurements for NLRP3. (**B**) (**b1**): Caspase-1 immunohistochemistry in cardiac tissue (20 X, scale bar 100 µm), (**b2**): Mean optical density readings for Caspase-1. (**C**) (**c1**): GSDMD immunohistochemical analysis in heart tissue (20 X, scale bar 100 µm), (**c2**): Mean optical density values for GSDMD. (**D**) (**d1**): NLRP3 immunohistochemical analysis in the blood vessel (20 X, scale bar 100 µm), (**d2**): NLRP3 average optical density measurement. (**E**) (**e1**): Caspase-1 immunohistochemical analysis in the blood vessel (20 X, scale bar 100 µm), (**e2**): Caspase-1 average optical density measurement. (**F**) (**f1**): GSDMD immunohistochemical analysis in the blood vessel (20 X, scale bar 100 µm), (**f2**): GSDMD average optical density measurement. *: *p* < 0.05, **: *p* < 0.01, compared with the Control group; #: *p* < 0.05, ##: *p* < 0.01, compared with the DBP-High group; NS: *p* > 0.05. *n* = 3.

For immunohistochemistry, due to limitations such as sample availability and ethical approval, a sample size of *n* = 3 was ultimately adopted. The determination of this sample size was based on the generally accepted standards in this field for similar studies [32,33,34]. The immunohistochemical analysis of pyroptosis (Figure 6) demonstrated that, in comparison to the Control group, the levels of NLRP3 and Caspase-1 in cardiac tissue were significantly elevated in both the DBP-Medium and DBP-High groups (Figure 6(a2,b2)). Concurrently, the level of GSDMD was notably increased in the DBP-High group (Figure 6(c2)). Additionally, in vascular tissue, Caspase-1 levels were significantly higher in the DBP-Medium and DBP-High groups compared to the Control group (Figure 6(e2)), while NLRP3 and GSDMD levels were markedly elevated in the DBP-High group (Figure 6(d2,f2)). These findings indicate that long-term exposure to high doses of DBP can induce the expression of pyroptosis-related proteins in vascular tissues. Furthermore, administration of vitamin E resulted in a significant reduction in pyroptosis-related protein levels in both cardiac and vascular tissues. These results suggest that oxidative inflammation in the heart and blood vessels induced by prolonged DBP exposure may be mediated by pyroptosis and can be mitigated by the antioxidant properties of vitamin E.

### 3.5. The Impact of Long-Term Exposure to DBP on Serum Metabolites in SD Rats

**Figure 7:**
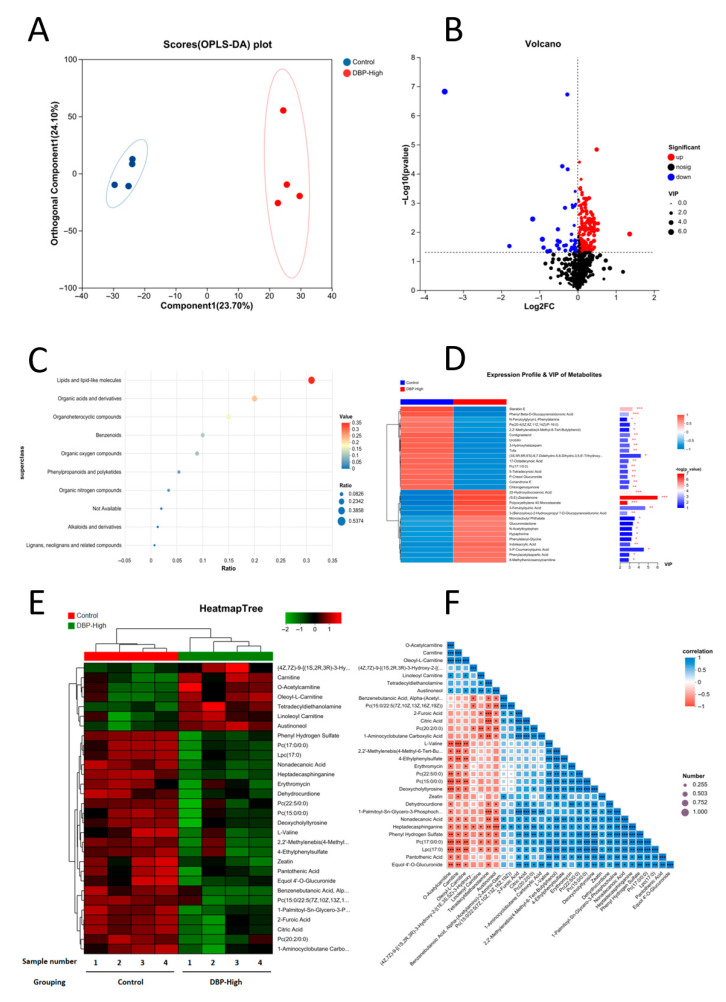
Impact on metabolic profile. (**A**) Score plot generated from OPLS-DA analysis for the Control and DBP-High groups. (**B**) Differentially identified metabolites found in the Control compared to the DBP-High groups. (**C**) Ratio of differential metabolites present in the Control and DBP-High groups. (**D**) VIP analysis and expression profiles of metabolites assessed between the Control and DBP-High groups. (**E**) Heatmap dendrogram for the Control and DBP-High groups. (**F**) Correlation analysis of metabolites between the Control and DBP-High groups. *: *p* < 0.05, **: *p* < 0.01, ***: *p* < 0.001.

Data accuracy assessment: We performed a non-targeted metabolomic on serum samples collected from SD rats in Control and DBP-High groups, utilizing the LC-MS detection platform. This analysis included 4 parallel samples from each group, leading to a total of 8 samples. The specific results of the OPLS-DA model test are included in the Supplementary Materials. The outcomes of Principal Component Analysis (OPLS-DA) conducted on the metabolite data were as follows: (1) There was robust parallelism observed within the Control and DBP-High groups, alongside statistically significant differences identified between the Control and DBP-High groups (Figure 7A); (2) Prolonged exposure to elevated levels of DBP had a considerable impact on the serum metabolites of SD rats, as demonstrated by the volcano plot, which revealed a reduced number of differential metabolites. The findings were distinct and well-defined, thus aiding in data analysis and the validation of experiments (Figure 7B).

Analysis of metabolite differences: Results from the HMDB database regarding compound classification showed that the main distinctive metabolites observed between the two sample groups included lipid and lipid-like molecules, organic acids along with their derivatives, and organoheterocyclic compounds (Figure 7C). To pinpoint possible biomarkers that set these two groups, we established a VIP > 1. The top 30 metabolites that fulfilled this criterion were then represented in a heatmap (Figure 7D). Notable variations in metabolites comprised Sterebin E, (S,E)-Zearalenone, and Polyoxyethylene 40 Monostearate, among others. Additionally, cluster detection of the metabolites (Figure 7E) showed that Nonadecanoic Acid, L-Valine, Zeatin, and Pantothenic Acid were significantly downregulated in the DBP-High group compared to the Control group. Furthermore, correlation analysis demonstrated that Nonadecanoic Acid, L-Valine, Zeatin, and Pantothenic Acid exhibited obvious positive correlations between each other (Figure 7F).

### 3.6. The Impact of Long-Term Exposure to DBP on KEGG Signaling Pathways Related to Metabolism in SD Rats

**Figure 8:**
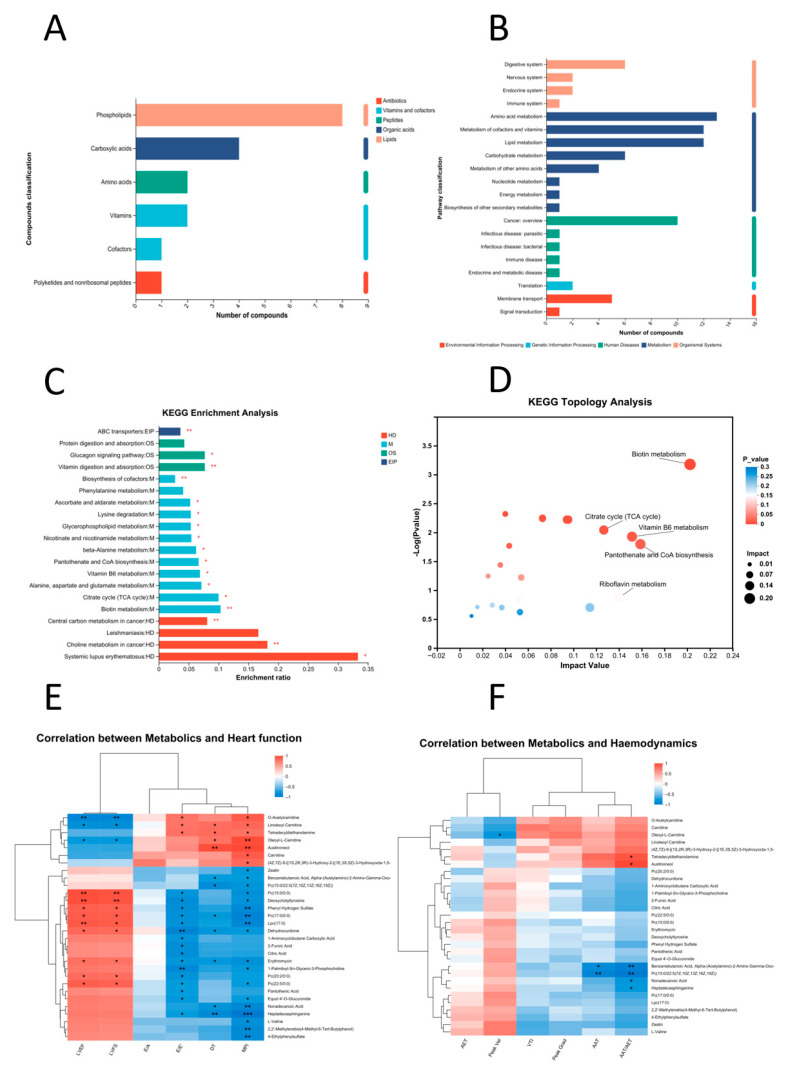
Impacts on pathways associated with metabolism according to KEGG. (**A**) Classification of compounds related to KEGG. (**B**) Categorization of KEGG pathways. (**C**) Analysis of KEGG enrichment. (**D**) Analysis of KEGG topology. (**E**) Correlation assessment between differential metabolites and cardiac function. (**F**) Correlation assessment between differential metabolites and hemodynamic parameters. *: *p* < 0.05, **: *p* < 0.01, ***: *p* < 0.001.

Metabolic pathway exploration: This study conducted a classification analysis of compounds related to KEGG (Figure 8A). The findings showed that the class of Phospholipids was the most prevalent. By mapping these distinctive metabolites to KEGG pathways, we uncovered direct links to pathways like Amino acid metabolism, Metabolism of cofactors and vitamins, and Lipid metabolism (Figure 8B). Utilizing a KEGG-centered strategy, we assessed metabolites that displayed significant variation among the groups, which allowed us to pinpoint the related metabolic pathways that were notably enriched due to these metabolites. The analyses of KEGG enrichment (Figure 8C) were primarily concentrated on human diseases and metabolism. Noteworthy pathways included Biotin metabolism, Citrate cycle, Vitamin B6 metabolism, Pantothenate and CoA biosynthesis, and Riboflavin metabolism (Figure 8D).

Metabolic correlation analysis: The analysis of the association between metabolites and cardiac function (Figure 8E) revealed that Nonadecanoic acid, L-valine, Zeatin, and Pantothenic acid are closely related to the levels of cardiac function indicators. These metabolites primarily exhibit positive correlations with LVEF and LVFS, while demonstrating negative correlations with the E/E’ ratio, DT, and MPI index. However, correlation analysis between metabolites and hemodynamic indicators (Figure 8F) indicated that the associations between metabolites and hemodynamic indicators were not particularly significant.

## 4. Discussion

DBP is a common environmental contaminant that can build up in living organisms, becoming more concentrated through the food chain and eventually impacting human health. Compelling evidence now suggests that DBP may induce CVD, including damage to the heart and blood vessels [9,10]. It is essential to enhance our understanding of the toxic cardiovascular effects of DBP and its mechanisms of action, particularly considering the increasing public health burden posed by cardiovascular diseases. In this study, we investigated the pathways through which long-term exposure to DBP induces cardiac and vascular injury and dysfunction. Additionally, we examined the association between DBP and metabolic pathways. Furthermore, we confirmed that the antioxidant vitamin E has a protective effect.

Our results reveal the complete pathological reaction chain: (1) DBP induces oxidative stress, leading to the overproduction of ROS; (2) the ROS produced by oxidative stress activate the NLRP3, which triggers NLRP3/Cas-1/GSDMD-dependent pyroptosis, resulting in oxidative inflammation; (3) DBP is closely associated with metabolic pathways, primarily including Biotin metabolism, Citrate cycle, Vitamin B6 metabolism, Pantothenate and CoA biosynthesis, and Riboflavin metabolism; (4) Under the influence of these multiple factors, DBP exerts cardiovascular toxicity, causing damage and dysfunction in the heart and blood vessels.

### 4.1. Evidence of the Cardiovascular Toxic Effects Exhibited by DBP

Evidence that DBP can influence heart function: It is well established that the LVFS and LVEF serve as key indicators of left ventricular systolic activity, playing a crucial role in the overall function of the heart [35]. In this study, echocardiographic analysis (Figure 3A) demonstrated a significant decrease in LVEF in the DBP-High group compared with the Control group (Figure 3(a2)), suggesting that DBP induces left ventricular systolic dysfunction. Recent research into diastolic heart failure has enhanced researchers’ interest in the indicators used for assessing left ventricular diastolic function. Commonly utilized mitral Doppler ultrasound indicators, such as E/A, E/E’, and DT, are employed for evaluating diastolic function [36]. Historically, the E/A ratio was the primary measure for assessing early left ventricular diastolic function; however, its susceptibility to various influencing factors can occasionally lead to pseudonormalization. Consequently, it is generally necessary to combine DT and E/E’ for a comprehensive evaluation of left ventricular diastolic function [37]. The echocardiographic findings from this study (Figure 3B) indicate that both DT and E/E’ were significantly elevated in the DBP-High group compared with the Control group (Figure 3(b2,b5)), suggesting that DBP may adversely affect cardiac diastolic function. The findings are similar to previous research results [38]. As cardiac function declines, the MPI index tends to increase. The findings of this study (Figure 3(b3)) reveal that the MPI in the DBP-High group is significantly greater than that in the Control group. These results corroborate the previously mentioned findings, indicating that DBP can adversely impact cardiac function.

Evidence that DBP can influence vascular function: Previous studies have demonstrated that hemodynamics is closely linked to vascular function [39,40]. The results of this study (Figure 3D) indicate that, compared with the Control group, the aortic arch wall echo in the DBP-High group was significantly enhanced, suggesting that DBP may lead to damage of the vascular intima. Additionally, the AAT and AAT/AET levels in the DBP-High group were significantly higher than those in the Control group, indicating that DBP may induce hemodynamic abnormalities. Collectively, these results suggest that long-term exposure to DBP may adversely affect vascular function, aligning with the findings of Stanic et al. [10].

Evidence that DBP can influence the pathology of cardiac and vascular tissues: Notably, the histopathological examination of the heart and blood vessels further corroborates these conclusions. The results of H&E staining (Figure 4A,C) indicated that, compared with the Control group, the myocardial and vascular endothelial cells in the DBP-High group were disorganized, with significant inflammatory infiltration in the interstitium, suggesting the presence of pathological damage. Furthermore, the results of Masson staining (Figure 4B,D) revealed a marked increase in collagen deposition in the cardiac and vascular tissues of the DBP-High group compared with the Control group. The increase in collagen deposition and fibrosis is a primary contributor to tissue stiffness and may exacerbate organ function [41]. These findings further confirm that DBP can induce cardiac and vascular damage.

### 4.2. Molecular Mechanism of Oxidative Inflammation Mediated by DBP

The leading role of oxidative stress: Our preliminary experiments demonstrate that exposure to DBP induces oxidative damage [13,14,15]. Based on this evidence, we hypothesize that the mechanism through which DBP exerts cardiovascular toxicity is linked to oxidative stress. We measured various indicators of oxidative stress, including ROS, MDA, and GSH. ROS is molecular oxygen intermediates produced during the reduction process. The accumulation of ROS leads to oxidative stress, resulting in tissue damage [42]. GSH serves as the primary scavenger of ROS, and its deficiency may reflect the extent of oxidative damage within the body. Excessive ROS can initiate lipid peroxidation by attacking lipid molecules, which alters the membrane fluidity and integrity of cells. This alteration increases the permeability of cell membranes and organelles, consequently affecting ion transport and energy metabolism through the cross-linking of membrane components [43]. MDA, a major metabolite of lipid peroxidation, serves as an indicator for assessing the extent and rate of lipid peroxidation. Our experimental results (Figure 5) indicate that DBP exposure elevates ROS and MDA levels in the heart and blood vessels while concurrently reducing GSH levels. Notably, recent studies have established a strong correlation between oxidative stress and pyroptosis [44,45,46]. Therefore, we speculate that the increase in ROS generation may contribute to the pyroptosis induced by DBP exposure. To test this hypothesis, we carried out experiments using the ROS scavenger known as Vitamin E. The findings revealed that the scavenging of ROS notably diminished the activation and cleavage of the NLRP3 inflammasome within the DBP-High group (Figure 6A,D), suggesting that ROS play a role in promoting DBP-induced pyroptosis. In conclusion, our results offer significant evidence that ROS serves as the main factor driving DBP-induced pyroptosis in cardiac and vascular cells.

The mediating role of pyroptosis in oxidative inflammation: Pyroptosis is an inflammatory form of cell death that is characterized by the activation of Caspase-1, which necessitates the presence of a protein platform known as the inflammasome [47]. Among the various inflammasomes, NLRP3 has been investigated extensively. When activated, NLRP3 brings in ASC (an apoptosis-associated speck-like protein that contains a CARD domain) and initiates the activation of Caspase-1. This process results in the release of inflammatory mediators, mainly IL-1β and IL-18, which ultimately incite an inflammatory response [48]. Studies have demonstrated that pyroptosis-mediated inflammatory responses are closely associated with cardiovascular diseases. Yue and colleagues discovered that pyroptosis mediated by NLRP3 worsens myocardial hypertrophy, fibrosis, and the dysfunction caused by pressure overload in mice [49]. Duewell et al. discovered that NLRP3-mediated pyroptosis is significantly related to the inflammatory characteristics of atherosclerosis [50]. Our study revealed that exposure to DBP induces pyroptosis in the hearts and blood vessels of SD rats, leading to the upregulation of pyroptosis-related proteins NLRP3, Caspase-1, and GSDMD (Figure 6), and ultimately increasing the release of inflammatory factors such as IL-1β and IL-18 (Figure 5). These findings suggest that pyroptosis-mediated inflammatory injury may be a crucial mechanism by which DBP induces cardiac and vascular damage. Our study provides new insights into the cardiovascular toxicity of DBP.

### 4.3. Various Effects of DBP on Metabolites in Rat Serum

Differences in the oxidative stress: Cluster analysis of metabolites (Figure 7E), L-Valine, Zeatin, and Pantothenic Acid were significantly down-regulated in the DBP-High group compared with the Control group. Studies have demonstrated that the metabolism of L-Valine, Zeatin, and Pantothenic Acid is closely associated with oxidative stress [51,52,53]. Furthermore, the KEGG topological s (Figure 8D) indicated that the TCA cycle and vitamin B6 metabolism were significantly enriched as differential metabolic pathways. Research indicates that pyruvate carboxylase enhances the antioxidant capacity by sustaining the citrate cycle and redox metabolism in the liver [54]. Additionally, vitamin B6 has been shown to alleviate cadmium chloride-induced oxidative stress-mediated memory deficits in the hippocampus of mice, with the specific mechanism potentially linked to the p-JNK/Nrf2/NF-kB signaling pathway [55]. These findings further substantiate that DBP can promote oxidative stress and emphasize the leading role of oxidative stress.

Differences in the pyroptosis pathway: Based on a cluster analysis of metabolites (Figure 7E), the levels of L-Valine were notably reduced in the DBP-High group in comparison with the Control group. Research suggests that the metabolism of L-Valine is strongly linked to pyroptosis [56]. Additionally, an analysis of KEGG topology (Figure 8D) revealed that the Citrate cycle was a pathway with significant differences. Research has demonstrated that melatonin can mitigate Castration-Resistant Prostate Cancer by modulating the pyroptosis pathway, with the underlying mechanism potentially linked to mitochondrial targeting, including effects on the Citrate cycle and oxidative phosphorylation (OXPHOS) [57]. These findings underscore the pivotal role of pyroptosis in the cardiovascular toxicity associated with DBP.

Differences in the inflammatory pathway: According to the cluster analysis of metabolites (Figure 7E), Nonadecanoic Acid, L-Valine, and Pantothenic Acid were significantly down-regulated in the DBP-High group compared with the Control group. Studies have demonstrated that the metabolism of Nonadecanoic Acid, L-Valine, and Pantothenic Acid is closely linked to the inflammatory response [58,59,60]. Furthermore, the KEGG topological analysis (Figure 8D) revealed significant enrichment in differential metabolic pathways, specifically in Pantothenate and CoA biosynthesis, as well as Riboflavin metabolism. Research indicates that isosteviol sodium can modulate glycerophospholipid metabolism and mitigate macrophage-driven inflammation, with its mechanism of action associated with Pantothenate and CoA biosynthesis [61]. Additionally, Zhang et al. found that the biomimetic remodeling of microglial riboflavin metabolism can enhance cognitive function by regulating neuroinflammation [62]. These findings underscore the critical role of inflammatory responses in the cardiovascular toxic effects induced by DBP.

Variations in metabolism and related diseases: Analysis of KEGG enrichment (Figure 8C) demonstrated a notable enrichment of distinct metabolic pathways. Notably, the pathways related to human diseases include Systemic lupus erythematosus, Choline metabolism in cancer, Leishmaniasis, and Central carbon metabolism in cancer. These findings further enhance our understanding of the toxicological effects of DBP.

### 4.4. The Protective Effect of Vitamin E

Studies have demonstrated that vitamin E is a potent antioxidant capable of effectively scavenging and neutralizing ROS [63]. The incorporation of vitamin E into this research has yielded two significant biological insights. First, vitamin E functions as an antioxidant, underscoring the leading role of oxidative stress in the process of pyroptosis. Second, it exhibits a protective effect against cardiac and vascular injury and dysfunction. Our study suggests that vitamin E may serve as both a preventive and therapeutic agent to mitigate DBP-induced cardiac and vascular injury and dysfunction in SD rats.

### 4.5. Innovation

This study is the first to elucidate the effects of long-term exposure to DBP on cardiac and vascular tissues, while experimentally validating the associated mechanisms. Three significant findings emerge from this research: (1) Long-term exposure to DBP can lead to damage in cardiac and vascular tissues, ultimately impairing organ function. (2) Inflammatory damage induced by ROS-mediated pyroptosis plays a key role in the cardiovascular toxic effects of DBP, with the antioxidant vitamin E serving as further evidence for the oxidative damage pathway. (3) Metabolomic analyses indicate that DBP exposure can disrupt multiple metabolic pathways, thereby enhancing our understanding of its toxicological effects.

Limitations in this study: This study acknowledges certain limitations. Due to the limitations of resources, the sample sizes used in the experiments such as echocardiography, immunohistochemistry and metabolomics in this study were relatively small. We will further increase the sample size in subsequent experiments to obtain more convincing results, and replace the LSD test with a more robust post hoc test. Regarding the functional interpretation of metabolites, we are merely at the speculative stage. We will conduct further verification in subsequent experiments.

## 5. Conclusions

In summary, this study confirms that long-term exposure to DBP may cause cardiovascular damage and functional abnormalities, further elucidating the underlying toxicological mechanisms. The results indicate that exposure to DBP can lead to damage in the heart and blood vessels, ultimately affecting organ function. This damage is accompanied by the activation of oxidative stress, pyroptosis pathways, inflammatory responses, and abnormal metabolic pathways. Additionally, vitamin E, recognized for its antioxidant properties, plays a significant ameliorative role in this context. Currently, knowledge regarding the toxic effects of DBP on cardiovascular diseases and its mechanisms of action remains limited. Therefore, the impact of DBP intake on cardiovascular function and human health should be prioritized as a primary area of concern.

## Figures and Tables

**Table 1:** Grouping and experimental treatments. “+” represents “add”, “--” represents “no add”.

Group ID	Group Names	Treatments for Different Groups			
		Gavage DBP 0.01 mg/kg/d	Gavage DBP 1 mg/kg/d	Gavage DBP 50 mg/kg/d	Gavage Vitamin E
Group 1	Control	--	--	--	--
Group 2	DBP-Low	+	--	--	--
Group 3	DBP-Medium	--	+	--	--
Group 4	DBP-High	--	--	+	--
Group 5	DBP-High + Vitamin E	--	--	+	+

## Data Availability

Data will be made available on request.

## Supplementary Materials

The following supporting information can be downloaded at: https://www.mdpi.com/article/10.3390/toxics13100815/s1, File S1: Metabolite Processing Result Table; File S2: Metabolomic.

## Abbreviations

AAT	Aortic acceleration time
AET	Aortic ejection time
Caspase-1	Cysteine-requiring aspartate protease-1
CVD	Cerebrovascular disease
DBP	Dibutyl phthalate
GSDMD	Gasdermin D
GSH	Glutathione
IL-18	Interleukin-18
IL-1β	Interleukin-1β
LVEF	Left ventricular ejection fraction
LVFS	Left ventricular fraction shortening
MDA	Malondialdehyde
MPI	Myocardial performance index
NLRP3	NOD-like receptor thermal protein domain associated protein 3
ROS	Reactive Oxygen Species
SD	Sprague-Dawley
TCA	Tricarboxylic acid
TD	Deceleration time
VTI	Velocity time integral
