# Supplementary material for: Probing the Cardiovascular Toxic Effects of Long-Term Exposure to Dibutyl Phthalate in Sprague-Dawley Rats Based on Oxidative Inflammation and Metabolic Pathways: Implications for the Heart and Blood Vessel
Source: Toxics. 2025 Sep 25;13(10):815. doi: 10.3390/toxics13100815 (PMC12567603; doi:10.3390/toxics13100815)
Supplement: Supplementary file 1 [file toxics-13-00815-s001.zip › File S2 - Supplementary materials.pdf]

Supplementary Materials

Article

Probing the Cardiovascular Toxic Effects of Long-Term Exposure to Dibutyl Phthalate in Sprague-Dawley Rats Based on Oxidative Inflammation and Metabolic Pathways: Implications for the Heart and Blood Vessel

Xiao Liang [1,2,†], Qi Huang [1,2,†], Yang Wu [1], Deyu Zhu [1], Zhuangzhuang Wei [1], Qing Feng [1,3], Ping Ma [1], Xu Yang [1,4], Cuiyu Bao [1] and Xinyu Bao [1,2,*]

[1] Key Laboratory of Environmental Related Diseases and One Health, Xianning Medical College, Hubei University of Science and Technology, Xianning 437100, China; lxssg123456@163.com (X.L.); hq18748714752@163.com (Q.H.); wysj2007@126.com (Y.W.); zdy1123a@163.com (D.Z.); wzzgood@126.com (Z.W.); fengqing123@163.com (Q.F.); mping68@126.com (P.M.); yang-xu@mail.ccnu.edu.cn (X.Y.); bcy_tiaopi@126.com (C.B.)

[2] School of Public Health and Nursing, Hubei University of Science and Technology, Xianning 437100, China

[3] Centre for Biological Science and Technology, Key Laboratory of Cell Proliferation and Regulation Biology of Ministry of Education, Faculty of Arts and Sciences, Beijing Normal University, Zhuhai 519000, China

[4] Institute of Natural Antioxidants and Antioxidant Inflammation, Dali University, Dali 671003, China

* Correspondence: bcy@hbust.edu.cn

† These authors contributed equally to this work.

Metabolomic

The specific sample preparation and instruments

The LC-MS analysis was conducted using the Thermo Fisher Scientific UHPLC-Exploris240 system, which integrates ultra-high performance liquid chromatography with Fourier transform mass spectrometry. The sample processing procedure is as follows: Precisely pipette 200 μL of the sample into a 1.5 mL centrifuge tube, add 800 μL of an extraction solution (methanol:acetonitrile = 1:1, v:v) containing four internal standards, including 0.02 mg/mL L-2-chlorophenylalanine. Vortex-mix for 30 seconds, followed by low-temperature ultrasonic extraction at 5°C and 40 kHz for 30 minutes, and then allow the mixture to stand at -20°C for 30 minutes. After centrifugation at 13,000 g and 4°C for 15 minutes, transfer the supernatant and dry it under a nitrogen flow. Reconstitute the residue with 120 μL of a resolubilization solution (acetonitrile:water = 1:1), vortex-mix again for 30 seconds, and perform low-temperature ultrasonic extraction at 5°C and 40 kHz for 5 minutes. Finally, centrifuge at 13,000 g and 4°C for 10 minutes, transfer the supernatant to an injection vial with an insert for instrumental analysis, and separately pipette 20 μL of supernatant from each sample to create a quality control sample. The chromatographic conditions were set as follows: An ACQUITY UPLC HSS T3 column (100 mm × 2.1 mm i.d., 1.8 μm; Waters, Milford, USA) was employed with mobile phase A consisting of 95% water and 5% acetonitrile (containing 0.1% formic acid), and mobile phase B comprising 47.5% acetonitrile, 47.5% isopropanol, and 5% water (containing 0.1% formic acid). The injection volume was 3 μL, with a column temperature maintained at 40°C. For mass spectrometric analysis, samples were ionized using electrospray ionization, and mass spectra signals were acquired in both positive and negative ion scanning modes.

Detailed Qualitative and Quantitative Information of LC Non-target Metabolomics

The raw data were imported into the processing software Progenesis Q v3.0 (Waters

Corporation, Milford, USA) for library search. The main databases included mainstream public databases such as http://www.hmdb.ca/ and https://metlin.scripps.edu/.

During the library search process: The signal-to-noise ratio (S/N) was ≥ 3. When the S/N ratio of the measurement results was higher than this threshold, it was considered that the result was reliable. Expression levels were retained; otherwise, the expression levels were empty.

Qualitative analysis: Based on the mass-to-charge ratio (m/z) of the parent ion in the primary mass spectrum, as well as possible adduct ions and isotope peaks, the molecular formula can be predicted. According to a mass deviation of 10 ppm, it can be matched with substances in the database to determine possible metabolites; The secondary spectra corresponding to each parent ion's daughter ions are matched with the possible substances in the database. Using an improved weighted mass cosine similarity for scoring, substances with a certain score value or above are selected as candidate matches (30 points).

Quantitative analysis: Using data processing software, the chromatographic peaks detected in the sample are integrated. The peak area of each characteristic peak represents the relative quantitative value of a metabolite. The quantitative results are standardized using the total peak area, and finally, the (quantitative) results of the metabolites are obtained.

Data Analysis: Perform data preprocessing. Preprocessing steps: Remove substances with more than 20% missing values in each group; Fill missing values with the minimum value (minimum value for each substance row); Normalize the sum (eliminate systematic errors in each stage); Filter by RSD (remove substances with relative standard deviation > 30%); Proceed with subsequent analysis after preprocessing.

Overview of the OPLS-DA Model

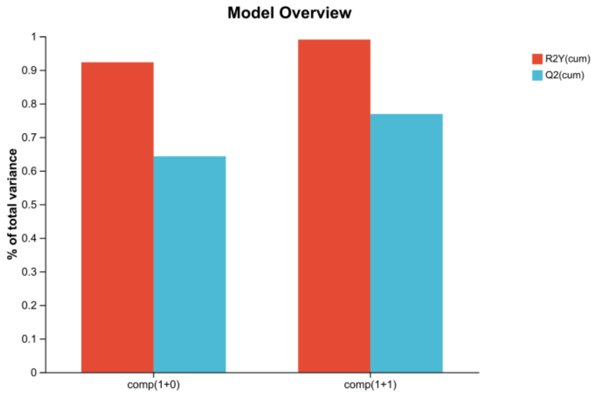

Note: Selection of the number of principal components in OPLS-DA. R2Y and Q2 are used to evaluate the modeling and prediction capabilities of the OPLS-DA model. The larger the cumulative values of R2Y and Q2Y, the more stable and reliable the model is.

OPLS-DA permutation test

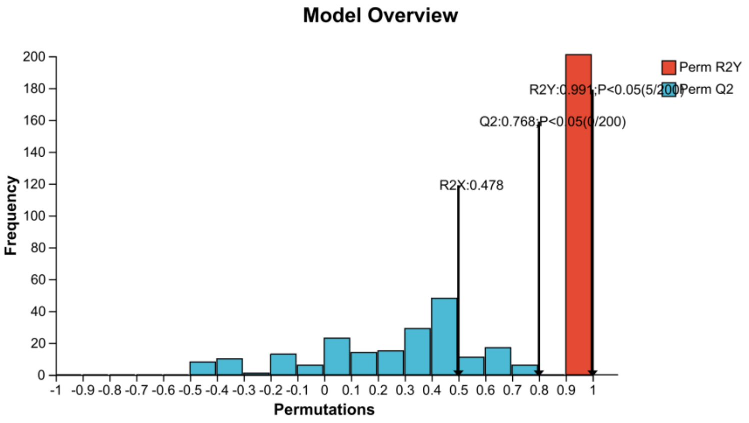

Note: The horizontal axis represents the accuracy rate of the permutation test random model, and the vertical axis represents the number of random models. The red bars represent the number of Q values obtained from the permutation test, and the blue bars represent the number of R2Y values obtained from the permutation test. The p-value = the number of random models that outperform the original model in the permutation test / the total number of random models in the permutation test. For example, for Q2, the p-value is 0.01, indicating that in the permutation test process, 1% of the random models have a better predictive ability than the original model; for R2Y, the p-value is 0.05, indicating that in the permutation test process, 5% of the random models have a better explanatory ability than the original model. Generally, it is considered that the model is optimal when the p-value is less than 0.05.

Model Parameter Table

A	R^2X	R^2X(cum)	R^2Y	R^2Y(cum)	Q^2	Q^2(cum)
p1	0.237	0.237	0.923	0.923	0.643	0.643
o1	0.241	0.478	0.0676	0.068	0.126	0.126
sum		0.478		0.991		0.768

Note: R2X and R2Y represent the explanatory rates of the established model for the X and Y matrices respectively; R2X(cum) and R2Y(cum) indicate the cumulative explanatory rates; Q2 indicates the predictive ability of the model. The closer these three indicators are to 1, the more stable and reliable the model is; p1 represents the principal component; o1 and o2 respectively represent the first and second orthogonal components.

The full identification and quantification details to support Figures 7 and 8

Figure 7A (Note: OPLS-DA score plot. The OPLS-DA score plot undergoes orthogonal rotation to eliminate information irrelevant to the grouping, thereby enabling better distinction of differences between groups and enhancing the efficacy of the model. Comp1 - first predicted principal component explained degree, orthogonal Comp1 - first orthogonal component explained degree.)

Figure 7B (Note: The x-axis represents the fold change value of the expression difference of metabolites between the two groups, which is log2FC. The y-axis represents the statistical test value of the expression change difference of metabolites, which is -log10(p_value). The higher the value, the more significant the expression difference. Both the x and y axis values have been logarithmically processed. Each point in the figure represents a specific metabolite, and the size of the point indicates the Vip value. By default, the red points represent metabolites with significantly upregulated expression, the blue points represent metabolites with significantly downregulated expression, and the gray points represent metabolites with no significant difference. The corresponding data can be found in the detail table of differences. After mapping all metabolites, it can be known that the points on the left represent metabolites with downregulated expression differences, and the points on the right represent metabolites with upregulated expression differences. The points closer to the left, right, top, and bottom have more significant expression differences.)

Figure 7C (Note: The horizontal axis represents Ratio, indicating the relative proportion of metabolite quantities in each category. The vertical axis represents each category. The color and size of the bubbles represent

the Ratio values.)

Figure 7D (Note: On the left side is the dendrogram of metabolite clustering, with closer branches indicating that the expression patterns of all metabolites within the sample are more similar; each column represents a sample, and below is the sample name; each row represents a metabolite, and the color indicates the relative expression level of the metabolite in the group of samples. The relationship between the color gradient and the numerical size is shown in the gradient color blocks. On the right side is the VIP bar chart of metabolites, the length of the bar represents the contribution value of the metabolite to the difference between the two groups. The default value is not less than 1, and the larger the value, the greater the difference of the metabolite between the two groups. The color of the bar represents the significance of the difference of the metabolite between the two groups, that is, the smaller the P_value, the larger the -log10(P-value), and the darker the color. The asterisk on the right represents: * represents $P < 0.05$, ** represents $P < 0.01$, and *** represents $P < 0.001$.)

Figure 7E (Note: Each column in the figure represents a sample, and each row represents a metabolite. The colors in the figure indicate the relative expression levels of the metabolites in the respective group of samples. The specific trend of the expression level changes can be seen in the numbers below the color bars on the right. On the left side is the dendrogram of metabolite clustering, and on the right side are the names of the metabolites. The closer the branches of two metabolites are, the more similar their expression levels are. On the top is the dendrogram of sample clustering, and on the bottom are the names of the samples. The closer the branches of two samples are, the more similar the expression patterns of all metabolites in these two samples are, that is, the expression trend of metabolites is more similar.)

Figure 7F (Note: On the right and bottom of the figure are the names of the metabolites, while on the left and top is the dendrogram of the metabolite clustering. Different colors represent the magnitude of the correlation coefficient. Positive and negative values indicate positive and negative correlations, respectively. The closer the absolute value is to 1, the higher the positive or negative correlation between the metabolites.)

Figure 8A (Note: The vertical axis represents the KEGG compound secondary classification category, and the horizontal axis represents the number of metabolites annotated to this category. Based on the biological functions of the metabolites, they are mainly classified into:Organic acids, Lipids, Carbohydrates, Nucleic acids, Peptides, Vitamins and Cofactors, Steroids, Hormones and transmitters and Antibiotics.)

Figure 8B (Note: The vertical axis represents the secondary classification of KEGG metabolic pathways, while the horizontal axis shows the number of metabolites annotated to this pathway. KEGG metabolic pathways can be divided into 7 major categories: Metabolism, Genetic Information Processing, Environmental Information

Processing, Cellular Processes, Organismal Systems, Human Diseases and Drug Development.)

Figure 8C (Note: The horizontal axis represents the pathway name, and the vertical axis represents the enrichment rate. This represents the ratio of the number of metabolites enriched in the pathway (Metabolite number) to the number of metabolites annotated to the pathway (Background number). The larger the ratio, the greater the degree of enrichment. The color gradient of the columns indicates the significance of enrichment. The darker the color, the more significantly the KEGG term is enriched. Markers of *** indicate Pvalue or FDR < 0.001, ** indicate Pvalue or FDR < 0.01, and * indicate Pvalue or FDR < 0.05.)

Figure 8D (Note: Each bubble in the figure represents a KEGG Pathway; the horizontal axis represents the relative importance of metabolites in the pathway, as indicated by the Impact Value; the vertical axis represents the significance of metabolite enrichment in the pathway, as indicated by -log10(Pvalue); the size of the bubble represents the Impact Value; the larger the bubble, the greater the importance of the pathway.)

Figure 8E and 8F (Note: On the right side of the figure are the names of metabolites, and at the bottom are the names of associated data. Each cell in the figure represents the correlation between two attributes (metabolite and associated feature), and different colors indicate the magnitude of the correlation coefficient between the attributes. Note: On the right side of the figure are the names of metabolites, and at the bottom are the names of associated data. Each cell in the figure represents the correlation between two attributes (metabolite and associated feature), and different colors indicate the magnitude of the correlation coefficient between the attributes. The asterisk represents the size of the significance p-value, * represents P < 0.05, ** represents P < 0.01, and *** represents P < 0.001.)

The identified metabolites

Metab ID	Metabolite
metab_336	Succinic Acid Semialdehyde
metab_387	4-Amino-3-Hydroxybutyric Acid
metab_389	Carnitine
metab_390	N(6)-Methyllysine

metab_ 491	Delta-Valerobetaine
metab_ 513	O-Acetylcarnitine
metab_ 530	8-Amino-7-Oxononanoic Acid
metab_ 536	Sterebin E
metab_ 594	N-Alpha-Acetyllysine
metab_ 618	1,2,3,4-Tetrahydro-3-Isoquinolinecarboxylic Acid
metab_ 631	1,4-Dihydro-1-Methyl-4-Oxo-3-Pyridinecarboxamide
metab_ 693	O-Succinyl-L-Homoserine
metab_ 741	Hydroxyphenylacetylglycine
metab_ 755	1-Acetamidocyclopentanecarboxylic Acid
metab_ 839	Phe Gly Leu
metab_ 893	Hypaphorine
metab_ 1054	Vitamin B7
metab_ 1079	4-Hydroxy-3-Methoxybenzoic Acid Ethyl Ester
metab_ 1109	Benzenebutanoic Acid, Alpha-(Acetylamino)-2-Amino-Gamma-Oxo-
metab_ 1120	3-(Benzoyloxy)-2-Hydroxypropyl ?-D-Glucopyranosiduronic Acid
metab_ 1298	1-[Ethyl-(6-Hydrazinylpyridazin-3-Yl)Amino]Propan-2-Ol
metab_ 1318	Coriandrone E
metab_ 1408	2-Hydroxyquinoline
metab_ 1445	Phenylalanyl-Glycine
metab_ 1526	2-Hydroxyphenethylamine
metab_ 1633	Equol

metab_ 1744	Ile-Ile-Ala-Glu-Lys
metab_ 1863	3-(4-Hydroxyphenyl)-3,5,6,8-Tetrahydro-2H-Chromene-4,7-Dione
metab_ 1906	Monoisobutyl Phthalate
metab_ 1920	10,20-Dihydroxyeicosanoic Acid
metab_ 1949	Avocadene
metab_ 1954	Tetradecyldiethanolamine
metab_ 1956	Polyoxyethylene 40 Monostearate
metab_ 1974	Nonadecanoic Acid
metab_ 2015	22-Hydroxydocosanoic Acid
metab_ 2017	Linoleoyl Carnitine
metab_ 2035	Oleoyl-L-Carnitine
metab_ 2482	Pc(22:5/0:0)
metab_ 2551	Pc(15:0/22:5(7Z,10Z,13Z,16Z,19Z))
metab_ 2564	Arachidyl Alcohol
metab_ 2641	1-Palmitoyl-Sn-Glycero-3-Phosphocholine
metab_ 2664	Deoxycholylasparagine
metab_ 2678	Pc(20:2/0:0)
metab_ 2831	Lysopc(22:5(7Z,10Z,13Z,16Z,19Z)/0:0)
metab_ 2861	Pc(17:0/0:0)
metab_ 2902	Sphingosine 1-Phosphate
metab_ 2905	Lysopc(15:0)
metab_ 2906	Lpc(17:0)

metab_3353	Pe(22:1/0:0)
metab_3583	Erythromycin
metab_3663	Pc(15:0/0:0)
metab_3669	Pc(17:1/0:0)
metab_3686	Lysope(0:0/22:5(4Z,7Z,10Z,13Z,16Z))
metab_3753	4-Hydroxy-2-Nonenal-[Cys-Gly] Conjugate
metab_3884	Ps(22:1(13Z)/22:5(7Z,10Z,13Z,16Z,19Z))
metab_3891	(4Z,7Z)-9-[(1S,2R,3R)-3-Hydroxy-2-[(1E,3S,5Z)-3-Hydroxyocta-1,5-Dien-1-Yl]-5-Oxocyclopentyl]Nona-4,7-Dienoylcarnitine
metab_3894	Koninginin E
metab_3900	P-Mentha-1,3,5,8-Tetraene
metab_4069	Austinoneol
metab_4084	Alizarin
metab_4087	8-Methylhenicosanoylcarnitine
metab_4206	Arachidoyl Ethanolamide
metab_4269	Hydroxytetradecadienyl-L-Carnitine
metab_4292	Heptadecasphinganine
metab_4297	Cortol
metab_4310	3-Indolepropionic Acid
metab_4356	Tanacetol B
metab_4398	Octanoyl-L-Carnitine
metab_4462	1-Naphthyl Beta-D-Glucuronide
metab_4475	Urobilin

metab_ 4488	(3S,5R,6R,6'S)-6,7-Didehydro-5,6-Dihydro-3,5,6'-Trihydroxy-13,14,20-Trinor-3'-Oxo-Beta,Epsilon-Caroten-19',11'-Olide 3-Acetate
metab_ 4579	Glucocorticoid Receptor Agonist
metab_ 5110	2-Quinolinecarboxylic Acid
metab_ 5111	N-Acetyl-Dl-Aspartic Acid
metab_ 5161	3-Methylhippuric Acid
metab_ 5171	Riboflavin
metab_ 5212	Monomethyl Phthalate
metab_ 5244	Di-O-Methylfraxetin
metab_ 5291	P-Acetaminobenzaldehyde
metab_ 5328	2-Methylbutyroylcarnitine
metab_ 5399	Zeatin
metab_ 5428	Delta-Valerolactam
metab_ 5476	Kynurenine
metab_ 5479	4-Quinolinecarboxylic Acid
metab_ 5525	N1-Methyl-2-Pyridone-5-Carboxamide
metab_ 5556	Beta-D-Glucosamine
metab_ 5568	Nipecotic Acid
metab_ 5651	Trigonelline
metab_ 5718	N2-Acetylornithine
metab_ 5746	Proline Betaine
metab_ 5753	Gly Pro
metab_	1-Aminocyclobutane Carboxylic Acid

5759	
metab_ 5775	2-Pyridineacetic Acid
metab_ 5803	L-Valine
metab_ 5836	Trimethylamine N-Oxide
metab_ 6184	L-Lysine
metab_ 6185	Pipecolic Acid
metab_ 6187	Hexamethyleneimine
metab_ 6931	L-Homocitrulline
metab_ 7093	2-Furoic Acid
metab_ 7095	Isocitric Acid
metab_ 7097	Citric Acid
metab_ 7116	Glutaconic Acid
metab_ 7275	4-Pyridoxic Acid
metab_ 7339	Porphobilinogen
metab_ 7505	Pantothenic Acid
metab_ 7735	Indoleacrylic Acid
metab_ 7747	4-Hydroxyphenyllactic Acid
metab_ 7839	Phenyl Beta-D-Glucopyranosiduronic Acid
metab_ 7880	3-Methoxyphenol Sulfate
metab_ 7884	Ab-Fubinaca Metabolite 3
metab_ 8078	Indole-3-Carboxilic Acid-O-Sulphate
metab_ 8079	Phenaceturic Acid

metab_ 8091	Phenylacetylaspartic Acid
metab_ 8157	8-Hydroxyquinoline
metab_ 8333	Ser Leu Ser Gly Leu
metab_ 8409	3-Feruloylquinic Acid
metab_ 8431	Chlorogenoquinone
metab_ 8484	P-Cresol Sulfate
metab_ 8531	Phenylpyruvic Acid
metab_ 8566	N-Acetyltryptophan
metab_ 8688	2-Propylglutaric Acid
metab_ 8775	Isopiperitenone
metab_ 8794	(1R,2R)-5,5-Dichloro-N-(1-Cyanocyclopropyl)-2-(4-(4-(Methylsulfonyl)Phenyl)-1-(2,2,2-Trifluoroethyl)-1H-Pyrazol-3-Yl)Cyclohexanecarboxamide
metab_ 8810	Epifisetinidol-(4Beta->8)-Catechin
metab_ 9065	6''-O-Malonyldaidzin
metab_ 9072	N-Feruloylglycyl-L-Phenylalanine
metab_ 9135	3-Hydroxyhalazepam
metab_ 9143	Parylene C
metab_ 9181	5-P-Coumaroylquinic Acid
metab_ 9488	Contignasterol
metab_ 9495	Sedanonic Acid
metab_ 9509	Pe(20:4(5Z,8Z,11Z,14Z)/P-16:0)
metab_ 9545	5-Tetradecynoic Acid
metab_	Phaseic Acid

9565	
metab_ 9570	Cavipetin C
metab_ 9592	1-Hydroxy-2-Naphthoate
metab_ 9610	Hernandulcin
metab_ 9611	Haloxyfop
metab_ 9696	17-Octadecynoic Acid
metab_ 9824	Pe(22:5/0:0)
metab_ 9825	Lysopc(20:5(5Z,8Z,11Z,14Z,17Z)/0:0)
metab_ 9826	(S,E)-Zearalenone
metab_ 9913	2,2'-Methylenebis(4-Methyl-6-Tert-Butylphenol)
metab_ 10136	Tauro-24,25-Dihydrofusidate
metab_ 10164	Buprenorphine
metab_ 10255	Lysopc(20:2(11Z,14Z)/0:0)
metab_ 10353	Deoxycholyltyrosine
metab_ 11382	Pe(17:0/0:0)
metab_ 11537	27-Nor-5B-Cholestane-3A,7A,12A,24,25-Pentol
metab_ 11572	Eupatorin
metab_ 11642	Ruscogenin
metab_ 11645	Tofa
metab_ 11745	Carnosol
metab_ 11835	Carboprost
metab_ 11846	N'-Nitrosoanabasine

metab_ 11851	Pe(20:3(6,8,11)-Oh(5)/Dime(13,5))
metab_ 11860	Dehydrocurdione
metab_ 11924	1-(4-O-Beta-D-Glucopyranosyl-3-Methoxyphenyl)-3,5-Dihydroxydecane
metab_ 12165	Enterolactone 3'-Sulfate
metab_ 12222	4-Ethylphenylsulfate
metab_ 12253	Glucuronolactone
metab_ 12370	Pelargonic Acid
metab_ 12492	2-Phenylethanol Glucuronide
metab_ 12502	Verbenalin
metab_ 12624	Mirificin
metab_ 12628	Foeniculoside X
metab_ 12640	Hesperetin 7-Glucoside
metab_ 12642	Equol 4'-O-Glucuronide
metab_ 12651	Naringenin 4'-O-Glucuronide
metab_ 12665	2-Hydroxyacetophenone Sulfate
metab_ 12962	2-Methylhippuric Acid
metab_ 12984	Hydroxy Tyrosol -Acetate
metab_ 13001	P-Cresol Glucuronide
metab_ 13198	(2-Oxo-2,3-Dihydro-1H-Indol-3-Yl)Acetic Acid
metab_ 13275	6-Hydroxy-1H-Indole-3-Acetamide
metab_ 13389	Malonic Acid
metab_ 13401	3-(3,5-Dihydroxyphenyl)-1-Propanoic Acid Sulphate

metab_ 13546	Blepharin
metab_ 13622	Phenyl Hydrogen Sulfate
metab_ 14088	5-Hydroxyindoleacetic Acid
metab_ 14230	2-Methylcitric Acid
metab_ 14420	2,4-Dihydroxybutanoic Acid
metab_ 14500	Threonic Acid
metab_ 15171	Cholylhistidine
